# 

**Published:** 1866-02

**Authors:** 


CONTENTS FOR FEBRUARY.

ORIGINAL CONTRIBUTIONS.

Valedictory Address delivered to the Graduating Class of Rush Medical College. By Prof. J. Adams Allen, M. D.......................... p. 49

Clinical Lectures on Diseases of the Eye. By E. L. Holmes, M. D........ 62

A Unique Case in Obstetrics. By E. Howard Irwin, M. D......... ........ 67

SELECTED ARTICLES........ 70

*Surgery*—Two Tumors removed from the Larynx in a Case of Aphonia of Six Years, followed by immediate Speech.

*Obstetrics*—On the Propriety of Inducing Premature Delivery.

*Materia Medica*—Nux Vomica.

*Miscellaneous*—The Cattle Plague; Homœopathic Trial in England—Dr. Kidd's Homœopathy—Anæsthesia—Modification in Canquoin's Caustic Paste.

EDITORIAL........ 87

Rush Medical College; Twenty-Third Anniversary—American Medical Association—Illinois State Medical Society—Cook County Hospital—Obituary, etc.

*Book Notices*—Obscure Diseases of the Brain and Mind—The Medical Record.

